# Assessing Oral Health Literacy in Children and Adolescents: Current Evidence, Measurement Gaps, and Developmental Considerations

**DOI:** 10.3390/children13101408

**Published:** 2026-10-15

**Authors:** Cansu Özşin Özler, Bahar Güçiz Doğan

**Affiliations:** 1 Department of Oral and Dental Health Research, Hacettepe University Graduate School of Health Sciences, Sihhiye, 06100 Ankara, Turkey; 2 Department of Pediatric Dentistry, Faculty of Dentistry, Hacettepe University, Sihhiye, 06100 Ankara, Turkey; 3 Institute of Public Health, Hacettepe University, Sihhiye, 06100 Ankara, Turkey; bdogan@hacettepe.edu.tr

**Keywords:** oral health literacy, health literacy, oral health behaviors, oral health outcomes, oral health disparities, oral health-related quality of life, children, oral health assessment

## Abstract

**Highlights:**

**What are the main findings?**

- Direct assessment of oral health literacy in children and adolescents is feasible, but current measures have important developmental and conceptual limitations.
- Existing measures mainly capture functional or cognitive skills rather than broader literacy competencies.

**What are the implications of the main findings?**

- Future tools should be developmentally appropriate and validated across age groups and cultures.
- Longitudinal studies are needed to determine how pediatric oral health literacy relates to later oral health outcomes.

**Abstract:**

Oral health literacy (OHL) is increasingly recognized as a multidimensional construct associated with oral health behaviors, healthcare utilization, clinical outcomes, and oral health-related quality of life. However, the strength and consistency of these associations vary across populations, outcomes, study designs, and measurement approaches, and much of the available evidence derives from adult populations. This narrative review examines current evidence on OHL-related outcomes with particular attention to children and adolescents and critically evaluates direct and caregiver-based assessment approaches. The review identifies a substantial developmental and measurement gap in pediatric OHL research. Studies involving children have frequently assessed parental or caregiver OHL rather than children’s own competencies, although these represent related but distinct constructs. Direct assessment of children and adolescents is feasible, but currently available instruments remain limited, have generally been evaluated within restricted age ranges and sociocultural settings, and predominantly emphasize functional or cognitive competencies. Broader dimensions such as communication, critical appraisal, navigation, decision-making, and application of oral health information are less consistently assessed. Developmentally appropriate OHL assessment should therefore account for cognitive and linguistic development, reading ability, communication skills, and increasing autonomy rather than simply adapting adult instruments. Future research should prioritize age-appropriate, culturally adaptable, and psychometrically robust measures that directly assess children’s and adolescents’ OHL across developmental stages and help clarify its relationships with oral health behaviors, service utilization, clinical outcomes, and oral health-related quality of life.

## 1. Introduction

The holistic perspective of health reflects its physical, mental, and social dimensions. Within this framework, oral health constitutes a fundamental component of general health and well-being and is closely linked to quality of life and essential functions such as eating, speaking, and social interaction. Beyond its local manifestations, poor oral health may have broader nutritional, psychosocial, and systemic implications, further emphasizing the importance of maintaining oral health across the life course [1].

Maintaining health depends not only on access to healthcare services but also on individuals’ ability to obtain, understand, evaluate, and use health-related information in everyday life. Health literacy therefore encompasses cognitive, social, and communication competencies required to navigate healthcare systems and make informed health-related decisions [2,3]. Oral health literacy (OHL) represents a context-specific extension of this concept and refers to the ability to obtain, understand, communicate, critically evaluate, and apply oral health-related information to support informed oral health decisions and behaviors [2,3,4,5,6,7]. Accordingly, OHL should be regarded as a multidimensional construct rather than a simple measure of oral health knowledge.

OHL has been associated with oral health behaviors, healthcare utilization, clinical outcomes, and oral health-related quality of life, although the magnitude and consistency of these associations vary across populations and study designs [6,8,9]. These findings should not be interpreted as uniformly independent or causal because OHL interacts with behavioral, socioeconomic, educational, familial, and healthcare system-related factors. OHL is therefore better understood as one component within a broader network of individual and contextual determinants of oral health.

The importance of oral health literacy may be particularly pronounced during childhood, when lifelong oral health behaviors, health beliefs, and health-related decision-making skills begin to develop [10,11,12]. Children’s oral health literacy evolves continuously with cognitive maturation, language development, educational experiences, and increasing autonomy [10,11]. Consequently, assessment approaches developed for adults may not adequately reflect children’s developmental characteristics or their evolving ability to understand and apply oral health information [11,13]. Given that childhood represents a critical developmental period during which oral health behaviors and literacy skills can be established and modified, identifying limited oral health literacy at an early age may provide opportunities for timely educational interventions before inadequate knowledge and behaviors persist into adolescence and adulthood [14,15].

Despite increasing recognition of the importance of OHL during childhood, available reviews indicate that OHL measurement instruments have predominantly been developed and validated in adult populations [16,17]. In contrast, relatively few instruments have been designed or validated for the direct assessment of children’s and adolescents’ own OHL [18,19,20], and pediatric studies have frequently relied on parental or caregiver OHL as an indirect indicator of the child’s oral health environment and healthcare decision-making context [21,22,23]. However, caregiver OHL and the child’s own OHL represent related but distinct constructs and should not be considered interchangeable. This imbalance highlights a substantial gap in the direct measurement of pediatric OHL.

This narrative review examines current evidence on oral health literacy in relation to oral health behaviors, clinical outcomes, healthcare utilization, oral health-related quality of life, and relevant social determinants, with particular attention to the extent to which this evidence applies to children and adolescents. It further critically reviews current approaches to OHL assessment in younger populations, including direct child and adolescent measures and caregiver-based approaches, and considers how developmental stage, cognitive and linguistic abilities, and increasing autonomy should inform age-appropriate assessment. Finally, the review identifies key measurement gaps and priorities for the development and validation of developmentally appropriate OHL instruments for children and adolescents.

## 2. Literature Search Approach

To support this narrative review, a focused literature search was conducted using PubMed/MEDLINE and Google Scholar for relevant literature published up to September 2026. Search terms included combinations of “oral health literacy,” “dental health literacy,” “child,” “children,” “adolescent,” “parent,” “caregiver,” “assessment,” “measurement,” “instrument,” “validation,” and terms related to oral health behaviors, clinical outcomes, dental service utilization, and oral health-related quality of life. PubMed/MEDLINE was used as the primary bibliographic database, while Google Scholar was used as a supplementary search source to identify additional relevant publications.

Studies were selected on the basis of their relevance to the conceptualization, measurement, and oral health-related correlates of OHL. Particular attention was given to studies that directly assessed OHL in children or adolescents, studies evaluating parental or caregiver OHL in relation to child oral health outcomes, and studies reporting the development or validation of OHL assessment instruments. Systematic and scoping reviews were preferentially used to characterize the broader evidence base, while original studies were included when they provided specific information on pediatric OHL instruments, developmental considerations, or findings not adequately represented in existing reviews. Studies focusing exclusively on general health literacy without clear relevance to oral health were not included in the core synthesis. The literature identification and selection approach was informed by recommended principles for transparent and methodologically rigorous narrative reviews [24,25]. The search was intended to provide a focused and transparent evidence base for narrative synthesis rather than an exhaustive systematic review.

## 3. Oral Health Literacy and Oral Health-Related Outcomes

Evidence linking OHL with oral health-related outcomes varies according to the population studied, the outcomes examined, and the approach used to assess OHL [9]. Importantly, studies assessing individuals’ own OHL should be distinguished from those evaluating parental or caregiver OHL in relation to children’s oral health outcomes. Accordingly, the available evidence is discussed separately for adults, parents and caregivers of children, and children and adolescents whose OHL has been assessed directly.

### 3.1. Evidence from Adult Populations

Studies involving adults have examined associations between individuals’ own OHL and oral health behaviors, clinical oral health status, dental service utilization, and oral health-related quality of life (OHRQoL). For example, Kanupuru et al. [26] investigated the relationship between OHL and oral health status among college students.

In a systematic review and meta-analysis including 25 studies, Firmino et al. [8] found no significant association between OHL and the frequency of dental visits among adults. Most included studies were cross-sectional (n = 21), and 17 were assessed as having a high risk of bias. Although higher OHL was associated with better oral health knowledge in several studies, the evidence regarding dental treatment-related outcomes, oral health behaviors, and oral health perceptions remained inconclusive.

Evidence concerning associations between OHL and clinical oral health conditions in adults remains inconsistent, with methodological and clinical heterogeneity limiting the interpretation of available findings [9].

Evidence regarding the relationship between health literacy and dental service utilization is also inconsistent. In a scoping review of 19 studies involving adults, Lu et al. [27] found that although some studies reported positive associations between health literacy and dental service utilization, the overall findings were inconsistent, and the underlying mechanisms remained unclear.

Beyond clinical indicators and healthcare utilization, OHL has also been examined in relation to oral health-related quality of life (OHRQoL), a multidimensional patient-reported outcome reflecting the physical, psychological, and social impacts of oral conditions. Evidence from adult populations [28,29,30] suggests that lower OHL may be associated with poorer OHRQoL. For example, a cross-sectional study among pregnant women found that lower OHL was independently associated with greater oral health-related impacts [28], while a study among university students also reported an association between OHL and OHRQoL although the strength of this relationship varied across participant groups [29]. These findings remain predominantly observational and should therefore be interpreted as associations rather than evidence of a direct effect of OHL on quality of life.

Overall, the available evidence suggests that adult OHL may be associated with selected oral health-related outcomes, particularly oral health knowledge and OHRQoL. However, inconsistent findings, methodological heterogeneity, and the predominance of observational study designs limit conclusions regarding its independent or causal contribution to clinical outcomes and dental service utilization.

### 3.2. Evidence from Parents and Caregivers of Children

In pediatric oral health research, parental or caregiver OHL has frequently been examined in relation to children’s oral health behaviors, clinical outcomes, dental service utilization, and OHRQoL, rather than directly assessing children’s own literacy competencies [22,31,32,33,34,35,36,37]. Sermsuti-Anuwat et al. [22] reported that although higher caregiver OHL scores in the “Economic Barriers” and “Utilization” domains were associated with a greater number of dental visits among foster children, overall caregiver OHL levels did not significantly influence foster children’s use of dental services. Dieng et al. [32] reported that the OHL level of Senegalese mothers was significantly associated with their children’s dental caries. Similarly, Ristow Montes et al. [33] found that untreated dental caries was more prevalent among preschool children whose caregivers had low OHL. Miller et al. [34] reported that caregivers of children with mild-to-moderate treatment needs were more likely to have higher OHL scores than those with severe treatment needs. Vann Jr. et al. [35] found that lower caregiver literacy was associated with deleterious oral health behaviors, including nighttime bottle use and no daily brushing/cleaning. Khodadadi et al. [37] found that inadequate parental OHL was associated with higher levels of dental caries and fewer dental fillings in children. In a systematic review comprising 10 cross-sectional studies, Firmino et al. [9] reported a weak association between lower OHL and dental caries in primary teeth; however, only three included studies reported findings for primary dentition. Zhang et al. [36] investigated the relationship between parental OHL and OHRQoL among preschool children and reported an association between parental OHL and children’s OHRQoL.

Taken together, caregiver-based studies suggest that parental or caregiver OHL may be associated with selected aspects of children’s oral health, including dental caries, preventive behaviors, dental service utilization, and OHRQoL. Nevertheless, the consistency and strength of these associations vary, and caregiver OHL should not be interpreted as a direct measure of children’s own oral health literacy.

### 3.3. Evidence from Direct Assessment of Children and Adolescents

Evidence directly linking children’s and adolescents’ own OHL with oral health-related outcomes remains limited. The Thai P-TOFHLiD validation study demonstrated the feasibility of directly assessing functional OHL among primary school children, although its associations with clinical oral health indicators were limited in the initial validation context [18]. In an Indonesian adolescent population, directly measured OHL was associated with the timing of the most recent dental visit [19].

More recently, Kamolchaiwanich et al. [38] directly assessed OHL among sixth-grade primary school students and reported associations between inadequate OHL and adverse clinical and behavioral outcomes, including untreated caries in permanent teeth, pulpal involvement of first permanent molars, poorer oral hygiene, and cariogenic snack consumption. However, associations were not consistently observed across all oral health indicators and behaviors.

Collectively, these studies suggest that directly measured pediatric OHL may be associated with selected oral health behaviors, dental service utilization, and clinical outcomes, including untreated dental caries and pulpal involvement. However, the limited number of studies, predominantly cross-sectional designs, restricted age ranges, and differences in measurement approaches preclude definitive conclusions regarding the consistency or predictive value of these associations across developmental stages and populations [18,19,38].

## 4. Oral Health Literacy, Social Determinants, and Oral Health Inequalities

Although OHL is commonly conceptualized in terms of individual knowledge and competencies, it is also shaped by broader social, economic, and environmental conditions. Educational attainment, socioeconomic circumstances, access to healthcare services, family resources, and social support may shape opportunities to obtain, understand, and use oral health information in everyday life [6,39,40]. These same contextual factors may also independently affect oral health behaviors, access to care, and oral health outcomes. OHL is therefore better understood as one component within a network of interacting individual, social, and structural determinants of oral health [6,22].

The relationship between oral health literacy and oral health inequalities should therefore be considered within broader life-course and socioecological frameworks that recognize the cumulative and interacting influences of individual, social, economic, and structural determinants on oral health [39,41]. Individuals with limited educational opportunities or restricted access to preventive healthcare may experience fewer opportunities to develop oral health literacy, while the same structural disadvantages may simultaneously limit access to oral healthcare services and increase the risk of adverse oral health outcomes. Consequently, oral health literacy may contribute to oral health inequalities as one component embedded within broader behavioral, social, and structural pathways. These interactions may become even more complex during childhood, when OHL develops through multiple and changing sources of information and support. In younger children, parents and caregivers play a central role by supervising daily oral hygiene, shaping dietary and dental-attendance practices, interpreting professional advice, and determining access to oral healthcare. As children progress through school and acquire greater autonomy, opportunities to develop OHL increasingly extend beyond the family environment to educational settings, oral healthcare encounters, and other social sources of health information. The extent and quality of these opportunities are also influenced by parental OHL, socioeconomic resources, educational exposure, and access to preventive dental care [6,40,42]. Pediatric OHL should therefore be understood as an evolving competency shaped through interactions between the child and the family, educational, healthcare, and broader social environments. Importantly, these environments influence but do not necessarily represent the child’s own OHL, particularly as autonomy and independent health-related decision-making increase with age. Recent evidence also highlights that oral health literacy operates within a broader social and structural context. Among Myanmar migrant families in Thailand [40], parents’ management of their children’s oral health was shaped not only by knowledge and information-seeking skills, but also by financial constraints, work schedules, language barriers, immigration-related challenges, and previous negative healthcare experiences. Similarly, qualitative findings from migrant mothers in Sweden [43] indicate that oral health literacy is closely linked to access to reliable information, trust in information sources, and the cultural appropriateness of dental care, underscoring the importance of considering social and contextual determinants alongside individual literacy skills. These findings suggest that disparities in oral healthcare utilization cannot be explained by individual oral health literacy alone but should also be understood in relation to wider social determinants and structural barriers. Menoncin et al. [44] found that children whose parents had higher OHL were more likely to have previously used dental services than those whose parents had lower OHL, suggesting an association between parental OHL and dental service utilization among preschool children.

From a public health perspective, childhood represents a unique opportunity for reducing future oral health inequalities. Oral health literacy is not a static characteristic but a developmental capacity that can be strengthened through age-appropriate education, supportive family and school environments, and accessible oral healthcare services. Identifying children with limited oral health literacy may provide opportunities for timely and developmentally appropriate educational support and may promote more favorable oral health behaviors and trajectories. However, whether strengthening OHL can reduce oral health inequalities remains uncertain and requires direct evaluation. Oral health inequalities are also strongly shaped by socioeconomic conditions, family resources, educational opportunities, accessibility of oral healthcare services, and other structural determinants. OHL should therefore be considered one potentially modifiable component within a broader network of influences rather than an independently established mechanism for reducing inequalities [14,15,39,41,45,46].

## 5. Assessment and Measurement of Oral Health Literacy

Approaches to OHL measurement have evolved in parallel with the conceptual expansion of health literacy. Early instruments largely focused on functional competencies such as word recognition, reading comprehension, and numeracy, whereas more recent approaches have attempted to capture broader dimensions including communication, information appraisal, decision-making, healthcare navigation, and application of oral health information in everyday contexts. This evolution is particularly relevant when considering the suitability of existing instruments for children and adolescents, whose literacy skills and health-related autonomy are still developing [47].

Accordingly, numerous instruments have been developed and validated for adult populations, including measures assessing functional, communicative, and critical dimensions of oral health literacy [48,49,50,51,52,53,54,55,56,57]. Examples include the Rapid Estimate of Adult Literacy in Dentistry (REALD-30) [55] which primarily assesses word recognition and therefore reflects functional literacy; the Test of Functional Health Literacy in Dentistry (TOFHLiD) [57] which evaluates reading comprehension and numeracy-related functional skills; and the Oral Health Literacy Instrument (OHLI) [52] which similarly emphasizes reading comprehension and numeracy. In contrast, the Health Literacy in Dentistry scale (HeLD), both short and the original form, refs. [48,51] was developed as a broader multidimensional measure encompassing domains such as communication, access, understanding, receptivity, utilization, support, and economic barriers. Similarly, OHL-AQ [50] was developed to measure functional OHL, with additional sections for evaluating listening and decision-making skills. The Comprehensive Measure of Oral Health Knowledge (CMOHK) [49] was developed to assess conceptual oral health knowledge as a component of OHL. Nevertheless, even among multidimensional instruments, communicative and critical OHL competencies are not assessed consistently across tools. These instruments have substantially advanced the assessment of OHL in adults and contributed to understanding its relationship with oral health outcomes. However, they were developed and validated in adult populations with relatively mature cognitive, linguistic, and health-related decision-making capacities, which limits their direct applicability to children and adolescents.

The assessment of OHL during childhood presents unique methodological challenges. Children’s ability to obtain, understand, evaluate, and apply oral health information evolves alongside cognitive, linguistic, emotional, and social development [10]. At the same time, the context of health-related decision-making changes with age: younger children largely depend on parents or caregivers, whereas older children progressively assume greater autonomy and responsibility for their oral health behaviors [10,11]. Consequently, oral health literacy in childhood should be regarded as a dynamic developmental capacity, and assessment approaches should be sensitive to age, communication skills, educational experience, cognitive maturity, and degree of autonomy [11,58].

Reflecting these developmental and methodological challenges, relatively few instruments have been used to directly assess oral health literacy among children and adolescents. These include the P-TOFHLiD [18] for primary school children, the Indonesian adolescent OHL questionnaire [19], and more recently, the BOHL-YQ [20] for older adolescents. Available measures differ in their target age groups, conceptual scope, administration format, respondent type, and the specific dimensions of OHL they assess. Some primarily capture functional or cognitive competencies, whereas broader communicative and critical dimensions are less consistently represented.

An important distinction should also be made between direct assessment of children’s OHL and caregiver-based assessment in pediatric oral health research. In many studies, instruments originally developed for adults have been administered to parents or caregivers, and their OHL scores have subsequently been related to children’s oral health behaviors or outcomes [22,23,34,35]. In contrast, the HKOHLAT-P was specifically developed to assess parental OHL within a pediatric dentistry context [21], and was subsequently adapted and validated in other languages and settings [59,60]. Although such caregiver-based measures provide important information about the family oral health environment and healthcare decision-making context, they assess parental or caregiver OHL rather than the child’s own OHL abilities and should therefore not be interpreted as equivalent to direct child assessment. To clarify these distinctions, the principal pediatric and adolescent OHL instruments discussed in this review are summarized in Table 1, including their target populations, respondents, administration formats, principal OHL domains assessed, psychometric properties, and major limitations.

As shown in Table 1, the instruments developed or adapted for direct pediatric OHL assessment differ in their target populations, conceptual scope, and developmental foundations. The P-TOFHLiD, developed in Thailand by adapting an instrument originally designed for older adults, primarily assesses functional OHL among primary school children [18]. The Indonesian adolescent OHL questionnaire [19] and the BOHL-YQ [20] extend direct assessment to adolescent populations, although their approaches and domains differ. Despite demonstrating acceptable psychometric properties in their respective validation settings, these instruments have been evaluated in relatively restricted age groups and sociocultural contexts, raising questions about their broader developmental applicability.

An important distinction emerges when considering the clinical relevance of the P-TOFHLiD. Although the initial validation study reported limited associations with clinical oral health indicators [18], a subsequent cross-sectional study using the same instrument identified significant associations between inadequate OHL and selected adverse clinical outcomes, including untreated dental caries and pulpal involvement of first permanent molars [38]. These findings suggest that directly measured pediatric OHL may be clinically relevant, but they do not establish longitudinal predictive validity. More broadly, the available validation studies provide evidence of measurement reliability and validity within specific populations, while leaving questions about generalizability across developmental stages and sociocultural contexts unresolved.

Beyond the limitations documented in validation studies, concerns regarding the developmental appropriateness of adult-derived instruments remain largely conceptual. Although existing studies demonstrate the feasibility of direct pediatric OHL assessment, further research is needed to determine whether these instruments adequately capture developmentally relevant competencies [10,11].

A further limitation concerns the dimensions of OHL captured by existing measures. Many instruments emphasize functional or cognitive competencies, including reading comprehension, word recognition, and interpretation of written oral health information [11,16]. Although these abilities are important components of OHL, broader competencies such as communication, critical appraisal, navigation of oral health services, decision-making, and application of oral health information in age-specific everyday contexts are less consistently assessed [16,17]. This mismatch between contemporary multidimensional conceptualizations of OHL and the domains captured by currently available instruments supports the need for measures developed specifically around the developmental characteristics of children and adolescents [11,16].

## 6. Developmental Considerations for Child and Caregiver OHL Assessment

The relative contribution of direct child assessment and caregiver-based assessment is likely to change across childhood and adolescence. Current evidence does not support rigid chronological cut-offs for determining when direct OHL assessment becomes appropriate. Rather, assessment should take into account cognitive and linguistic development, reading ability, educational experience, prior exposure to oral healthcare, and the degree of autonomy in oral health-related behaviors and decision-making [10,11,58].

In younger school-age children, direct assessment may reasonably focus on concrete and developmentally accessible competencies, such as recognition of basic oral health concepts, understanding of simple preventive instructions, identification of appropriate oral health behaviors, communication of symptoms or needs, and knowing when and from whom to seek help. At this stage, reading-intensive assessment may underestimate OHL by conflating oral health literacy with general reading proficiency. Short, concrete, low-reading-burden items and, where appropriate, visual, scenario-based, or interviewer-assisted approaches may therefore be preferable [10,11,58]. Because caregivers remain responsible for many aspects of oral healthcare during this period, including dental attendance, supervision of oral hygiene, and treatment-related decisions, caregiver OHL remains highly relevant. Nevertheless, caregiver-based assessment should be considered complementary to, rather than a substitute for, direct assessment of the child’s own competencies [21,31,59,60,61].

During later childhood and early adolescence, direct assessment can progressively incorporate the ability to locate and understand oral health information, communicate with dental professionals, interpret preventive or treatment-related instructions, and apply information to everyday oral health decisions. Available pediatric OHL studies demonstrate that direct assessment is feasible among primary school children and adolescents [18,19,20]. During this transitional stage, parallel assessment of child and caregiver OHL may be particularly informative because responsibility for oral health behaviors and healthcare decisions is often shared.

In adolescence, as autonomy and independent health-information seeking increase, direct assessment should assume a more central role. In addition to functional comprehension, relevant competencies may include communication, appraisal of the credibility and relevance of oral health information, navigation of oral healthcare services, participation in treatment decisions, and application of information to increasingly independent health-related choices [10,11,13,58]. Caregiver OHL may remain relevant as a contextual factor but should not be interpreted as a proxy for the adolescent’s own OHL.

Accordingly, the choice between child-only, caregiver-only, or parallel child–caregiver assessment should be guided by the developmental context and the specific research question. Where responsibility for oral health is shared, parallel assessment may help distinguish limitations related to the child’s developing competencies from those related to caregiver literacy or the broader family and healthcare environment.

As summarized in Table 2, developmentally appropriate OHL assessment requires alignment between instrument content, administration format, and children’s evolving cognitive, linguistic, and decision-making competencies. In younger children, assessment should emphasize accessible and concrete oral health concepts while minimizing unnecessary reading demands. As children progress toward adolescence, greater attention can be given to communication, information appraisal, healthcare navigation, and increasingly independent oral health-related decisions. These considerations highlight the need for flexible, developmentally responsive approaches to instrument selection and adaptation [10,11,13,58].

Importantly, chronological age alone should not determine instrument selection or item difficulty. Children of the same age may differ substantially in reading proficiency, cognitive maturity, communication ability, educational exposure, previous contact with oral healthcare, and degree of autonomy. Content validity and comprehensibility should therefore be examined within the intended developmental group, ideally through age-appropriate pretesting or cognitive interviewing, rather than assuming that an instrument validated in one pediatric age group will perform equivalently across childhood and adolescence.

Although parental and caregiver OHL plays an important role in shaping children’s oral health behaviors, healthcare access, and treatment-related decisions, it does not necessarily reflect children’s own literacy competencies [21,31]. As children develop greater cognitive, communicative, and decision-making abilities, their capacity to understand, evaluate, and apply oral health information may increasingly differ from that of their caregivers. Direct assessment may therefore provide complementary information about children’s developing competencies and help identify literacy-related needs that caregiver-based measures alone may not capture [18,19]. Where responsibility for oral health is shared, combining child and caregiver assessments may offer a more comprehensive understanding of the individual and contextual factors influencing oral health-related decisions. This complementary approach may also inform the development of more targeted and developmentally appropriate oral health promotion strategies.

Future research should evaluate whether developmentally appropriate OHL measures can inform targeted educational interventions and improve the assessment of oral health promotion programs. Whether such approaches can contribute to reducing oral health inequalities remains uncertain and requires further investigation.

This review has several limitations. As a narrative review, the literature search was designed to provide a focused and transparent synthesis rather than an exhaustive systematic identification of all available studies, and relevant publications may therefore have been missed. In addition, no formal systematic risk-of-bias or certainty-of-evidence assessment was undertaken at the review level. These limitations are particularly important given the small number of studies directly assessing OHL in children and adolescents, the predominance of cross-sectional designs, and the heterogeneity of populations, instruments, and outcomes. Accordingly, the conclusions regarding pediatric OHL assessment and its associations with oral health-related outcomes should be interpreted as a critical synthesis of the current evidence rather than as definitive estimates of effect or causality.

## 7. Conclusions

Current evidence indicates that direct assessment of OHL in children and adolescents is feasible, but available instruments remain limited in developmental coverage, conceptual breadth, and validation across populations and cultural settings. Future instrument development should involve children and adolescents in item generation and content validation, incorporate functional, communicative, critical, navigational, and decision-making competencies, and include cognitive interviewing and testing across developmental stages, reading levels, and sociocultural contexts. Longitudinal research is also needed to establish responsiveness and predictive validity and to clarify how directly measured pediatric OHL relates to subsequent oral health behaviors, healthcare utilization, clinical outcomes, and oral health-related quality of life.

## Figures and Tables

**Table 1:** Principal oral health literacy instruments used for direct assessment of children and adolescents or caregiver assessment in pediatric oral health contexts.

Instrument	Year	Target Population/Age	Respondent	Administration Format	Main Domains Assessed	Main Psychometric Findings/Evidence of Associations with Outcomes	Key Limitations
Hong Kong Oral Health Literacy Assessment Task for Paediatric Dentistry (HKOHLAT-P) [21]	2013	Parents/caregivers of children in pediatric dentistry	Parent/caregiver	Task-based assessment	Recognition/naming, literacy/numeracy, comprehension; predominantly functional OHL	Cronbach’s α = 0.71; ICC = 0.63; convergent and predictive validity supported	Does not directly assess the child’s own OHL; evaluates parental OHL specifically within a pediatric dentistry context
Indonesian adolescent OHL questionnaire [19]	2015	Adolescents aged 12–14 years	Adolescent	Self-administered questionnaire	Direct adolescent OHL assessment, with emphasis on functional/cognitive components	Cronbach’s α = 0.77; ICC = 0.75; convergent and discriminant validity supported	Evaluated within a restricted age range and single cultural/educational setting; broader multidimensional OHL competencies are less clearly represented
Test of Functional Health Literacy in Dentistry for Primary School Children (P-TOFHLiD) [18,38]	2022	Grade-six primary school children, approximately 11–14 years	Child	Direct task-based/reading-comprehension assessment	Mainly functional OHL, particularly reading comprehension and interpretation of oral health information	Initial validation (2022): Cronbach’s α = 0.808; concurrent and convergent validity demonstrated. Subsequent study (2025): significant cross-sectional associations with selected clinical oral health outcomes.	Adapted from an adult instrument; limited coverage of broader OHL competencies. Clinical associations differed between studies, and longitudinal predictive validity remains unestablished.
Brazilian Oral Health Literacy-Youngs Questionnaire (BOHL-YQ) [20]	2026	Adolescents aged 15–19 years	Adolescent	Self-administered questionnaire	Cognitive and comprehension/decision-making domains	Acceptable internal consistency and structural validity reported	Derived from an adult OHL questionnaire rather than developed de novo specifically around adolescent developmental characteristics

**Table 2:** Proposed developmental framework for direct and caregiver-based oral health literacy assessment in children and adolescents.

Developmental Stage	Competencies to Assess	Item and Administration Characteristics	Role of Caregiver Assessment
Younger school-age childhood	Basic recognition and comprehension; preventive behaviors; communication of symptoms or needs; help-seeking	Short, concrete, low-reading-burden items; visual, scenario-based, or interviewer-assisted approaches where appropriate	Highly relevant for healthcare access, supervision, and treatment-related decisions; complementary to child assessment
Later childhood/early adolescence	Information access and interpretation; communication with professionals; application to everyday decisions	Age-appropriate written material; practical scenarios; comprehension and application tasks; increasing direct self-report	Parallel child–caregiver assessment may be particularly informative because responsibility is shared
Adolescence	Functional, communicative, and critical OHL; source appraisal; navigation; decision-making; independent application	Direct self-administered or task-based assessment; appraisal and decision-making items; more complex real-life scenarios	Contextual rather than substitutive; should not replace adolescent self-assessment

*Note: This proposed framework was informed by developmental health literacy models and measurement literature in children and adolescents, together with available studies of direct pediatric OHL assessment [10,11,13,18,19,58]. The stages are intended as a conceptual guide rather than rigid chronological age categories.*

## Data Availability

No new data were created or analyzed in this study. Data sharing is not applicable to this article.
